# Empirically Derived Dietary Patterns in UK Adults Are Associated with Sociodemographic Characteristics, Lifestyle, and Diet Quality

**DOI:** 10.3390/nu10020177

**Published:** 2018-02-06

**Authors:** Katharine Roberts, Janet Cade, Jeremy Dawson, Michelle Holdsworth

**Affiliations:** 1 School of Health and Related Research, University of Sheffield, Sheffield S1 4DA, UK; J.F.Dawson@sheffield.ac.uk (J.D.); michelle.holdsworth@sheffield.ac.uk (M.H.); 2 Health Improvement Directorate, Public Health England, London SE1 6LH, UK; 3 School of Food Science and Nutrition, University of Leeds, Leeds LS2 9JT, UK; J.E.Cade@leeds.ac.uk

**Keywords:** dietary patterns, diet quality, National Diet and Nutrition Survey, UK, socio-demographic

## Abstract

The aim of this study was to examine empirical dietary patterns in UK adults and their association with sociodemographic characteristics, lifestyle factors, self-reported nutrient intake, nutrient biomarkers, and the Nutrient-based Diet Quality Score (NDQS) using National Diet and Nutrition Survey data 2008–2012 (*n* = 2083; mean age 49 years; 43.3% male). Four patterns explained 13.6% of the total variance: ‘Snacks, fast food, fizzy drinks’ (SFFFD), ‘Fruit, vegetables, oily fish’ (FVOF), ‘Meat, potatoes, beer’ (MPB), and ‘Sugary foods, dairy’ (SFD). ‘SFFFD’ was associated positively with: being male; smoking; body mass index (BMI); urinary sodium; intake of non-milk extrinsic sugars (NMES), fat and starch; and negatively with: age; plasma carotenoids; and NDQS. ‘FVOF’ was associated positively with: being non-white; age; income; socioeconomic classification (National Statistics Socio-economic Classifications; NSSEC); plasma carotenoids; intake of non-starch polysaccharides and polyunsaturated fatty acids. It was negatively associated with: being male, smoking, BMI, urinary sodium, intake of saturated fat; and NMES and NDQS. Whilst the patterns explained only 13.6% of the total variance, they were associated with self-reported nutrient intake, biomarkers of nutrient intake, sociodemographic and lifestyle variables, and the NDQS. These findings provide support for dietary patterns analyses as a means of exploring dietary intake in the UK population to inform public health nutrition policy and guidance.

## 1. Introduction

The identification of underlying, consistent patterns of dietary intake is valuable in nutritional epidemiology [1]. Whilst looking at single or a few dietary components remains an important focus for nutritional epidemiological studies, this approach has some conceptual and methodological limitations [2]. Individuals do not purchase or consume dietary components, nutrients or, frequently, foods, as single items in isolation. Individuals usually consume multiple nutrients in one food item and a number of food items as part of one meal. The combinations of foods and meals consumed day to day may also be habitual. This has implications for public health nutrition policy and guidance, as focusing on single nutrients (e.g., saturated fat) or foods (e.g., free sugars) may not be easy messages for the general public to translate into dietary behaviour change. In addition, it is likely that diseases such as cancer are influenced by multiple biochemical and physiological interactions between nutrients and foods. Other substances within food with no nutritive value, such as phytochemicals, may also be influential in disease development or protection [3,4] and biochemical and metabolic interactions in the body between micronutrients, dietary components, and foods may complicate or confound attempts to identify associations between foods, nutrients, and disease [2,5]. These types of limitations may lead to misleading or erroneous conclusions about the relationship between a single nutrient, dietary component, or food with a particular health outcome [6].

Dietary patterns analyses overcome some of these limitations by treating diet as a multidimensional exposure, more reflective of free-living individuals’ habitual eating behaviours. These methods have been used widely to investigate and identify typologies of ‘whole diet’ and their association with a range of health outcomes in multiple settings and population groups. Dietary patterns analyses can be applied a priori in order to investigate associations with a pre-defined, theory based dietary pattern or a posteriori in order to identify empirical dietary patterns in populations. The latter requires analysis of dietary intake data using statistical methods such as factor analysis, cluster analysis, and reduced rank regression [7].

A priori dietary patterns analyses involve the development of a composite measure, score, or index to summarise the extent to which an individual adheres to a pre-defined dietary pattern based on existing dietary guidelines or known or hypothesised diet-disease associations [8,9]. The Mediterranean Diet [9,10,11], the U.S. ‘Healthy Eating Index’ (HEI) [12,13,14,15], and the ‘Dietary Approaches to Stop Hypertension’ (DASH) diet [16,17] are examples of a priori or theoretically-derived dietary patterns that have been demonstrated in epidemiologic studies to be associated with health outcomes such as risk of cardiovascular diseases and type 2 diabetes. Studies exploring dietary patterns and health using a posteriori methods have reported associations between identified dietary patterns and colorectal cancer [18], hypertension [19] coronary heart disease [20], body mass index (BMI) and obesity [21], and waist circumference [22]. In the UK, many studies of this type have been undertaken using data from the Avon Longitudinal Study of Parents and Children (ALSPAC), the Southampton Women’s Survey, the Low Income Diet and Nutrition Survey (LIDNS), and the British Prospective Cohort. Findings from dietary patterns studies using these datasets in the last decade include: an association between energy dense dietary patterns and childhood obesity [23,24]; an association between dietary patterns of men, socio-economic measures and nutrient intake [25]; poor diet in early childhood may be associated with small reductions in intelligence quotient (IQ) in later childhood [26]; an association between dietary patterns and educational attainment in children [27]; an association between energy density of diet in middle life with breast density 15 years later [28]; and a ‘prudent’ diet may protect against impaired lung function and chronic obstructive pulmonary disease [29].

Nutritional epidemiology is currently heavily reliant on self-reported dietary intake data due to the complexity of assessing what individuals consume, particularly on a large scale. Self-reported dietary intake data are subject to systematic measurement error, in particular relating to under-reporting of energy intake [30,31,32]. This has led to polarised views regarding the validity of this type of data [33,34,35]. Those who continue to value self-reported methods recognise that whilst these data are subject to error, they are an important source of, otherwise unavailable, insight into the dietary intake of populations for the purpose of informing public health policy [35]. In addition, ongoing work to mitigate these limitations through improvements and refinements to methods of data collection, dietary assessment, and statistical analysis provides opportunities to consider self-reported dietary data in the context of each individual study and to apply appropriate adjustments to optimise their validity and utility [32,36,37,38,39,40,41,42]. Where under-estimation of energy intake is not of primary importance, such as in investigations of dietary patterns, analyses of trends, or assessment of diet quality, then the use of self-reported dietary intake data is widely considered to be appropriate [35,43,44].

Investigations of dietary patterns are thus invaluable for public health nutrition policy and research, as they demonstrate the importance of focusing on total diet in policy, guidance, and interventions, as opposed to single dietary components. This study explored empirical dietary patterns in UK adults and their associations with sample characteristics, nutrient intake, and diet quality.

## 2. Materials and Methods

### 2.1. Dataset

The data used in the analyses were from the UK National Diet and Nutrition Survey (NDNS) Rolling Programme. Computerised files from the NDNS were obtained from the UK Data Service archives (www.ukdataservice.ac.uk, accession number 64197). The NDNS is an annual cross-sectional survey undertaken in the UK since 2008. Dietary intake data are gathered from a self-reported unweighed 3- or 4-day food diary. Blood plasma and urine are also collected from a consenting sub-sample approximately 8 weeks after the self-reported data collection, from which biomarkers of nutritional status are derived. For the purposes of the analyses here, all dietary data collected are assumed to be representative of habitual dietary intake and nutrient status. 

The NDNS contains data on more than 7000 foods, which are aggregated into 59 ‘main’ food groups and then disaggregated from these ‘main’ categories into ‘subsidiary’ food groups. The ‘main’ food groups categorise all dietary intake data. Each food code has a value for 54 nutrients including energy, sugars, vitamins, and minerals [45]. Table 1 lists the 60 food group variables included in the principal component analysis (PCA). All ‘main’ NDNS food groups were included in the analyses with the exception of ‘Commercial Toddlers Foods and Drinks’, which was irrelevant for the adult sub-sample, and ‘Dietary sweeteners’ as these data were not provided in the dataset. ‘Subsidiary’ food groups for ‘low fat spreads’, ‘reduced fat spreads’, and ‘miscellaneous’ were included due to their widely varying nutritional composition and contribution to levels of consumption of key nutrients such as non-milk extrinsic sugars (NMES) and polyunsaturated fatty acids (PUFA) [45]. Food groups were presented as average grams consumed daily by each individual.

Data were included in the analyses from years 1 to 4 of the NDNS (2008–2012) comprising a sample of 2083 adults (aged ≥ 19 years, mean age 49 years) [46]. In line with other studies of the NDNS, the full sample was included in the analyses, as it was not considered possible to separate under-reporters from under-consumers, for example, those who were unwell [47]. 

### 2.2. Statistical Analyses

A number of statistical analysis methods, such as cluster analysis and factor analysis, can be employed to undertake a posteriori dietary patterns analyses. Exploratory factor analysis and, in particular, principal component analysis (PCA) has been used in many studies to reduce large sets of dietary intake variables representing the total diet into smaller sets of variables and to identify underlying ‘dietary patterns’ [7,48]. For this study, PCA was used to derive the dietary patterns, as this method uses the degree to which foods are correlated with each other to derive a new, smaller set of composite variables [48]. These composite variables are uncorrelated with each other and therefore can be considered as representative of discrete dietary patterns. The extracted sets of composite variables are ‘components’ and the variables within them are ‘factors’. PCA produces as many ‘components’ as there are variables, but the first few that explain the largest proportion of the variance in the data are selected. All analyses were conducted in SPSS Version 22 statistical software package.

**Figure 1:**
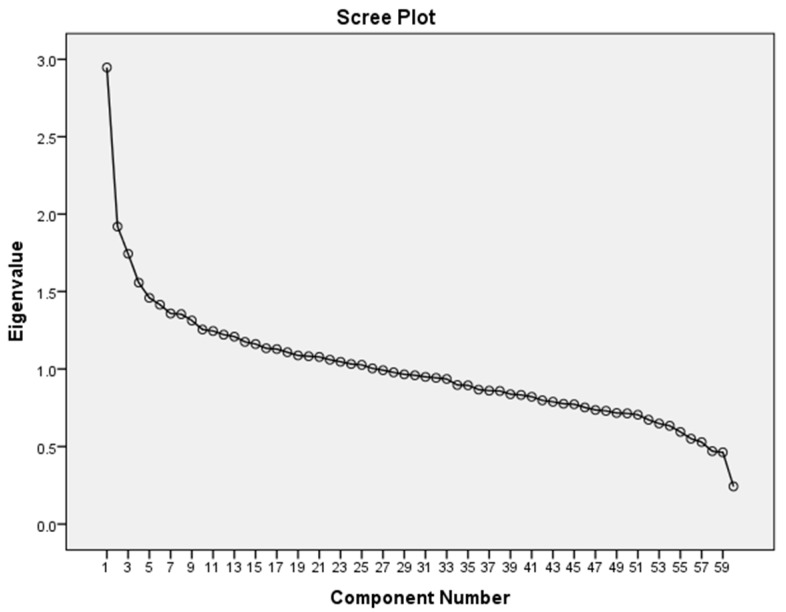
Scree plot for Principal Component Analysis of 60 food variables (National Diet and Nutrition Survey 2008–2012).

Prior to conducting the PCA, the Kaiser-Meyer-Olkin (KMO) measure and Bartlett’s test of sphericity were undertaken to ensure that the data were suitable for factor analysis. The KMO reached the acceptable limit of 0.5 [49] and Bartlett’s test of sphericity was significant, indicating that correlations between items were sufficiently large to undertake PCA. The 60 pre-defined food variables (average grams consumed per day) shown in Table 1 were entered into the PCA. Orthogonal rotation (varimax) was applied. The purpose of applying the rotation is that it redistributes the explained variance for each component and thus achieves a simpler structure [50]. As per convention for this type of analysis, the number of components selected was based on a visual assessment of the scree plot (Figure 1) and those with eigenvalues above the values of approximately 1.5, in order to identify the fewest number of patterns that explained the largest proportions of variance [7].

The ‘dietary patterns’ generated were characterised by high and low consumption of particular foods and drinks. All foods and foods groups have an associated factor loading with each component or ‘dietary pattern’. Foods and food groups with associations of ≥0.25 or ≤−0.25 were considered ‘moderate’ and ≥0.3 or ≤−0.3 were considered ‘strong’ or ‘significant’ contributors to a dietary pattern in line with previous studies [50,51,52]. 

Main effects regression analysis identified the associations between the dietary patterns and sociodemographic characteristics, lifestyle factors, self-reported intake, and biomarkers of single nutrients and overall diet quality. The following variables were included in the ‘sociodemographics and lifestyle’ model: age, household income (standardised to adjust for the number of individuals in the household), BMI (kg/m^2^), sex (male/female), ethnicity (white/non-white), National Statistics Socio-economic Classifications (NSSEC) (1—Higher managerial, administrative, and professional occupations; 2—Lower managerial, administrative, and professional occupations; 3—Intermediate occupations; 4—Small employers and own account workers; 5—Lower supervisory and technical occupations; 6—Semi-routine occupations; 7—Routine occupations; 8—Never worked and long-term unemployed), and smoking status (smoker/non-smoker). BMI and household income were included in the model as continuous variables in order to avoid the problems that have been demonstrated to result from artificial stratification [53]. For the variables sex, ethnicity, and smoking status, the category with the largest number of subjects was selected as the reference group. For NSSEC, the ‘never worked’ category was selected as reference as a comparator [54]. The following variables were included in the ‘nutrient intake and biomarkers’ model: self-reported intake per 1000 kcal of vitamins C, D, E, B_6_, B_12_, iron, folate, and magnesium; % food energy from non-milk extrinsic sugars (NMES), saturated fat, total fat, *n*-3 polyunsaturated fats (PUFA), *n*-6 polyunsaturated fats, non-starch polysaccharides (NSP); biomarkers from blood and urine samples of vitamin C, 25-hydroxy vitamin D, retinol, ferritin, triglycerides, total cholesterol, urinary sodium, and total plasma carotenoids.

Overall ‘diet quality’ was measured using the Nutrient-based Diet Quality Score (NDQS): a composite measure scoring levels of consumption of 12 nutrients and alcohol. The score was developed to reflect UK Dietary Reference Values (DRVs) and government dietary guidelines [55,56,57]. Inclusion of items and scoring was developed to take account of a priori knowledge of key nutrients for health and prevention of diseases with a focus on those diet-related issues that are most prevalent in the UK as well as population level nutrient deficiencies or over-consumption (for example, salt and saturated fat) [58,59,60,61]. The score was validated against biomarkers of nutrient intake derived from blood and urine samples [62]. Regression analysis was used to explore associations between the dietary patterns and the NDQS. Plots of residuals were visually assessed for evidence of homoscedasticity, constancy of variance, and outliers. Where variables were not normally distributed, regressions including the square of the independent variables were carried out to test for evidence of curvilinearity. Where the coefficient of the squared term was significant (thus indicating curvilinearity), the coefficients for both the value and the squared values were plotted to visually assess for potential curvilinear (quadratic) effects.

## 3. Results

Study population characteristics are described in Table 2. The mean age of the sample was 49 years and there were significantly more females than males and white than non-white participants.

### 3.1. Principal Component Analysis of Dietary Patterns

The first four principal components that explain the largest proportions of variance in the dietary intake data individually have eigenvalues above 1.5, and following a visual assessment of the scree plot (Figure 1), were selected and retained as ‘dietary patterns’. Together, the four components explain 13.6% of the total variance in the dietary intake data (3.9%, 3.7%, 3.1%, and 2.8%, respectively). Table 3 shows the rotated solution of the PCA. Foods with moderate or strong factor loadings were interpreted as those characterising each dietary pattern. The patterns were labelled subjectively, for ease of translation, based on these foods: ‘Snacks, fast food, fizzy drinks’ (SFFFD), ‘Fruit, vegetables, oily fish’ (FVOF), ‘Meat, potatoes, beer’ (MPB,) and ‘Sugary foods, dairy’ (SFD). Table 4 illustrates the foods with moderate/strong factor loadings for each of the four components or dietary patterns.

### 3.2. Regression Analyses

#### 3.2.1. Associations between Dietary Patterns and Sociodemographic Characteristics and Lifestyle Factors

Table 5 shows the main effects of each dietary pattern for sociodemographic characteristics and lifestyle factors. The majority of the associations were as might be expected. The SFFFD pattern was positively (*p* ≤ 0.05) associated with being male (0.24), being a smoker (0.16), and BMI (0.13) and was negatively associated with age (−0.03). The FVOF pattern was negatively associated with being male (−0.08), being a smoker (−0.37), and BMI (−0.002). This pattern was positively associated with being non-white (0.72) and with age (0.002). There was a clear gradient for the FVOF pattern with NSSEC, which was significant for all categories, with a lower NSSEC, such as never worked, being negatively associated with this pattern and a higher NSSEC, such as higher managerial and professional occupations, being positively associated this pattern. The SFD pattern was positively associated with being male (0.19), with age (0.003), and with all categories of occupation other than routine occupations. The SFD pattern was most strongly associated with lower supervisory and technical and semi-routine occupations (0.53 and 0.52, respectively, *p* < 0.01). It was negatively associated with being a smoker (−0.21), being non-white (−0.46), and with BMI (−0.02), which is contrary to the relationship that might be expected for this dietary pattern. The MPB pattern was significantly positively associated with being male (0.63), age (0.01), and being a smoker (0.21) and negatively with being non-white (−0.56). 

#### 3.2.2. Associations between Dietary Patterns and Nutrient Intake and Diet Quality

Table 6 shows the main effects of each dietary pattern for nutrient intake derived from self-reported dietary intake diaries and nutritional biomarkers derived from urine and blood plasma samples. The FVOF pattern was positively associated with intake per 1000 calories of vitamins C, D, E, B_12_, B_6_, iron, folate, and magnesium; proportion of food energy from *n*-3 and *n*-6 PUFAs; fibre (NSP) and biomarkers of vitamin C, D (25-hydroxy vitamin D), and A (retinol), iron (ferritin), and total carotenoids. It was negatively associated with the proportion of food energy from NMES, saturated fat, total fat, starch, and urinary sodium. The SFFFD pattern was negatively associated with intake per 1000 calories of vitamins C, D, E, B_6_, B_12_, folate, magnesium, proportion of food energy from *n*-3, intake of fibre (NSP), and biomarkers of vitamins C, D, A, total cholesterol, and total carotenoids. This pattern was positively associated with the proportion of food energy from NMES, total fat, *n*-6 PUFA, starch, and with urinary sodium. The MPB dietary pattern was negatively associated with intake of vitamins C and E, iron, and magnesium and with biomarkers for total carotenoids. It was positively associated with the proportion of food energy from NMES, saturated fat, total fat, and fibre (NSP) and plasma biomarkers of triglycerides and ferritin and urinary sodium. The SFD pattern had very similar associations to that of the SFFFD pattern, with the exception of being positively associated with the proportion of food energy from saturated fat and intake of fibre (NSP) and negatively associated with intake of *n*-6 PUFA, starch and biomarkers of retinol, ferritin and urinary sodium.

Table 7 shows the main effects of the NDQS for each dietary pattern unadjusted and adjusted for: age, gender, NSSEC, household income, ethnicity, smoking status, BMI, and total energy intake. All patterns were significantly predictive of diet quality as measured by the NDQS (*p* < 0.001). In both models, the SFFFD and MPB patterns were negatively associated with the NDQS, but the effect was slightly attenuated in the SFFFD pattern in the adjusted model compared with the unadjusted model. The SFFFD pattern was more strongly negatively predictive of the NDQS than the MPB pattern, with an expected decrease in NDQS score of 3.7 with every incremental increase in SFFFD factor score, compared with an expected decrease in NDQS score of 1.2 with every increase in MPB factor score, when all things remained equal. The effect of the MPB pattern on the NDQS was strengthened in the adjusted model (a 1.8 decrease on the NDQS compared with a 1.2 decrease). The FVOF and the SFD patterns were positively associated with the NDQS in both models; the FVOF pattern was more strongly positively predictive of the NDQS than the SFD. In the unadjusted model, for every incremental increase in FVOF score, the expected NDQS increase was 4.5 and for SFD it was 2.4. The effects of both patterns were slightly attenuated in the adjusted model compared with the unadjusted (with expected increases in NDQS of 3.8 and 1.3, respectively, compared with 4.5 and 2.4 in the unadjusted model).

## 4. Discussion

Analysis of dietary patterns is an important method in nutritional epidemiological research, as it allows diet to be explored and investigated as a multi-dimensional exposure, which more accurately reflects the way that free living individuals consume food. Nutrients and foods are rarely consumed in isolation, but as part of meals and habitual patterns of consumption. The aim of this study was to explore empirical patterns in UK adults and their associations with sociodemographic characteristics, lifestyle factors, self-reported intake, and biomarkers of nutrients and overall diet quality. Four patterns explained 13.6% of the total variance: ‘Snacks, fast food, fizzy drinks’ (3.9%), ‘Fruit, vegetables, oily fish’ (3.7%), ‘Meat, potatoes, beer’ (3.1%), and ‘Sugary foods, dairy’ (2.8%). Individuals scoring higher on the SFFFD pattern, which might also have been labelled as the ‘unhealthy’, ‘processed’, or ‘Western’ dietary pattern, were more likely to be male, white, a smoker, have a higher BMI, consume a greater proportion of food energy from (non-milk extrinsic) sugars, total fat, starch, and *n*-6 PUFA, and have higher urinary sodium levels. This pattern was negatively associated with age, self-reported intake per 1000 kcal, and biomarkers of a range of key nutrients, food energy from *n*-3 PUFA, intake of fibre (NSP), total plasma carotenoids (which are an indicator of fruit and vegetable intake [64]), total cholesterol, and a composite diet quality score calculated from self-reported dietary intake data (NDQS). The FVOF pattern, which might also have been labelled as the ‘healthy’ or ‘prudent’ diet was almost the inverse of the SFFFD pattern in its associations, with the exceptions of also being negatively associated with proportion of food energy from saturated fat, positively associated with biomarkers of 25-Hydroxy Vitamin D, retinol (Vitamin A), ferritin (iron), and having no significant association with total cholesterol. The MPB pattern, which could also be categorized as a ‘traditional British’ diet and the SFD or ‘sweet tooth’ pattern were both positively associated with being male, white, and older; with consuming a higher proportion of food energy from NMES, saturated fat, total fat, and fibre (NSP). Both patterns were negatively associated with intake per 1000 kcal of vitamin C, vitamin E, iron, magnesium; consuming a higher proportion of food energy from starch; and with total plasma carotenoids. There were some differences between these two patterns in that the SFD pattern was also negatively associated (where the MPB pattern had no significant association) with self-reported intake per 1000 kcals of vitamin D, B_12_, and folate, proportion of food energy from both *n*-3 and *n*-6 PUFA, and biomarkers for vitamin A. MPB was also positively associated with being a smoker, urinary sodium, and plasma ferritin (iron), where SFD was the inverse. MPB was also associated with plasma triglycerides, where SFD had no significant association. The differences in nutrient intake, urine and plasma levels of nutrients, and nutrient biomarkers are reflected in the differences in the foods that characterize each of these patterns. For example, the MPB pattern is characterized by processed red meat, which is high in salt and iron. Notably, three of the four dietary patterns, none of which represent a high quality diet, were more likely to be consumed by white males.

The findings suggest that there are proportions of the UK adult population that have patterns of dietary intake that are of varying dietary quality and are associated not only with demographic characteristics but also with lifestyle factors and socioeconomic measures. This is important, as lifestyle factors (such as smoking status) and socioeconomic measures (such as income and NSSEC) are associated with health outcomes independently of diet [65]. These findings support the use of empirically derived patterns as a method for exploring and describing dietary intake in the UK to inform public health nutrition policy and research. In addition, these data suggest that particular foods and other variables such as smoking status may be useful as proxies for dietary patterns in nutritional epidemiological studies. The use of the theory driven Nutrient-based Diet Quality Score also highlights the importance of these methods of analyses as a means to exploring diet as a multi-dimensional exposure. Therefore, this study demonstrates the usefulness of dietary patterns analyses methods, both empirically derived and a priori defined.

The results of this study are likely to be generalisable to the UK population. The NDNS is a robust dataset containing detailed dietary intake data from a nationally representative sample of UK adults. The methods for data collection, recruitment of participants, processing, and analysis of the data, including sourcing and updating food composition data, have been developed with close scrutiny and oversight from the commissioning departments in government [58]. PCA is a widely used method in nutritional epidemiological studies to reduce the detailed complexity of dietary intake into reduced sets of variables that represent ‘patterns’ of consumption that can be labelled for easy interpretation [48]. In addition, the dietary patterns identified in this study reflect those of other similar studies undertaken in the UK, which supports their generalisability and external validity [25,50,66] Studies undertaking PCA on data from the LIDNS [6] and the ALSPAC datasets [50] have reported numbers and types of dietary patterns similar to those identified in this study. PCA of the LIDNS dataset resulted in four dietary patterns explaining 16.5% of the total variance that were labelled as ‘fast food’, ‘health aware’, ‘traditional’, and ‘sweet’ [6]. Similarly, a study analysing dietary patterns in pregnant women in the ALSPAC dataset identified five dietary patterns explaining 32.7% of variance that were also similar to those identified in this study, which were described as: ‘health conscious’, ‘traditional’, ‘processed’, ‘confectionery’, and ‘vegetarian’. PCA in men in the same dataset identified four dietary patterns which were labelled similarly as: ‘health conscious’, ‘traditional’, ‘confectionery/processed’, and ‘semi-vegetarian’ [25]. 

There are a number of limitations to this study. The four identified dietary patterns explained only 13.6% of the total variance in the dietary intake data, which is a smaller proportion than other studies undertaking similar types of analyses in the UK [50,51,66,67,68]. This finding was potentially a result of the inclusion of a greater number of variables in the PCA than these other studies [69]. Studies with a lower number of dietary variables included in the PCA have resulted in a greater proportion of the variability explained [70]. Where food group categories are broad, foods that are weakly associated with a pattern may be classified in the same category as foods more strongly associated, thus increasing the amount of information captured by a specific pattern. This in turn may have an impact on the sensitivity of the components and thus their associations with disease or other variables [69]. Therefore, in some studies greater granularity may be more important in extracting the patterns than the amount of variance explained. This may be why some authors do not report the proportion of variance explained by the factors [48]. Another limitation is that some of the food group categories pre-defined in the NDNS dataset that were included in the PCA such as ‘yoghurt, fromage frais, and dairy desserts’ include a broad range of foods with widely varying nutritional compositions and impacts on health. The use of the NDQS as a composite measure of diet quality is both a strength and a limitation. The NDQS is a 13-item construct, based on UK DRVs, developed to score sensitively to current UK public health priorities and validated against nutrient biomarkers [62]. However, as with all such scores, decisions regarding the definition of ‘diet quality’, the inclusion of items, scoring ranges, and weighting were made with some level of subjectivity.

## 5. Conclusions

The findings in this study contribute to the current understanding of dietary intake in UK adults and have implications for the way that population level dietary intake is assessed and evaluated to inform public health policy and guidance. The findings show that empirically derived dietary patterns in UK adults are associated with sociodemographic characteristics, lifestyle, and diet quality, as measured by single nutrient indicators (both self-reported and biomarkers) and a composite Nutrient-based Diet Quality Score. They also suggest that there are combinations of foods and other sociodemographic or lifestyle variables that could be explored as proxies for dietary patterns in nutritional epidemiological studies and dietary assessment. The dietary patterns identified in this study are similar to some of those identified in other UK studies where different datasets, methods of data collection, and population subgroups have been utilised. This supports their validity despite only a relatively low proportion of total variance in the dietary intake data being explained. This is a significant finding for public health policy, as it highlights the importance of focusing on the ‘whole diet’ in exploring population level diet, targeting interventions, and developing public health messages as opposed to single foods or nutrients. The findings are also significant for public health researchers, as they provide significant support for the use of dietary patterns analyses as valid and insightful methods for exploring dietary intake and habits in the UK population.

## Figures and Tables

**Table 1:** List of UK National Diet and Nutrition Survey (NDNS) food variables included in the principal components analysis (PCA). PUFA: polyunsaturated fatty acids.

Food Group Variable	
1. 1% milk	31. High fibre breakfast cereals
2. Beef, veal, and dishes	32. Lamb and dishes
3. Butter	33. Liver products and dishes
4. Other margarine, fats, and oils	34. Meat pies and pastries
5. Other milk and cream	35. Nuts and seeds
6. PUFA margarine oils	36. Oily fish
7. Semi skimmed milk	37. Other bread
8. Whole milk	38. Other meat and meat products
9. Wholemeal bread	39. Pasta rice and other cereals
10. Ice cream	40. Pork and dishes
11. White fish coated or fried	41. Salad and other raw vegetables
12. Beer, lager, cider, and perry	42. Sausages
13. Crisps and savoury snacks	43. White bread
14. Fruit juice including smoothies	44. Yogurt, fromage frais, and dairy desserts
15. Soft drinks (low calorie)	45. Other breakfast cereals
16. Soft drinks (not low calorie)	46. Other potatoes, potato salads and dishes
17. Spirits and liqueurs	47. Other white fish, shellfish, fish dishes
18. Sugar confectionery	48. Vegetables not raw
19. Tea coffee and water	49. Chicken and turkey dishes
20. Wine	50. Puddings
21. Bacon and ham	51. Brown granary and wheat germ bread
22. Biscuits	52. Skimmed milk
23. Buns, cakes, pastries, and fruit pies	53. Sugars, preserves, and sweet spreads
24. Burgers and kebabs	54. Dry weight beverages *
25. Cheese	55. Low fat spread not polyunsaturated **
26. Chips (fried), roast potatoes, potato products	56. Low fat spread polyunsaturated **
27. Chocolate confectionery	57. Reduced fat spread not polyunsaturated ***
28. Coated chicken and turkey	58. Reduced fat spread polyunsaturated ***
29. Eggs and egg dishes	59. Savoury sauces, pickles, gravies, condiments *
30. Fruit	60. Soup homemade and retail *

* Subsidiary food group ‘Miscellaneous’; ** Subsidiary food group ‘Low fat spread’; *** Subsidiary food group ‘Reduced fat spread’.

**Table 2:** Study population characteristics: National Diet and Nutrition Survey 2008–2012 (*n* = 2083).

Characteristics	*n*	%	Mean (Standard Deviation)
Males	901	43.3	
Females	1182	56.7	
Age (years)			49 (17)
White	1877	91.5	
Non-white	168	8.5	
NSSEC Group			
Higher managerial and professional	309	14.9	
Lower managerial and professional	553	26.6	
Intermediate occupations	191	9.2	
Small employers	222	10.7	
Low supervisory and technical occupations	203	9.8	
Semi-routine occupations	289	13.9	
Routine occupations	235	11.3	
Never worked	43	2.1	
Smoker	488	24	
Non-smoker	1557	76	
Equivalised household income (£)	1777		32,035 (23,570)
BMI (kg/m^2^)	1902		27.7 (5.4)
Total energy intake diet only (kcal)	2083		1803 (575)

NSSEC: National Statistics Socio-economic Classifications; BMI: body mass index.

**Table 3:** Rotated PCA solution: food variables and factor loadings for each retained principal component (Dietary Pattern).

Food Variables (Average g/Day)	Principal Component (Dietary Pattern)			
	1 (SFFFD)	2 (FVOF)	3 (MPB)	4 (SFD)
Soft drinks not low calorie	0.51	0.03	0.10	0.03
Crisps and savoury snacks	0.48	−0.01	0.00	0.24
Soft drinks low calorie	0.42	0.11	−0.07	0.07
Coated chicken and turkey	0.40	−0.02	−0.07	0.06
Tea, coffee, and water	−0.38	0.20	0.05	0.25
Burgers and kebabs	0.37	−0.08	0.004	−0.01
High fibre breakfast cereals	−0.35	0.09	−0.05	0.14
Chips, fried and roast potatoes, and potato products	0.32	−0.28	0.25	0.07
Wholemeal bread	−0.28	0.16	−0.08	0.16
Sugar confectionery	0.27	0.18	−0.05	0.24
Pasta, rice, and other cereals	0.26	0.19	−0.20	−0.05
Soup homemade and retail	−0.20	0.06	−0.04	0.02
Chicken and turkey dishes	0.17	0.14	−0.12	−0.09
Low fat spread polyunsaturated	−0.06	0.03	0.03	0.03
Fruit	−0.30	0.56	−0.12	0.19
Salad and other raw vegetables	−0.14	0.51	−0.05	0.02
Yogurt, fromage frais, and dairy desserts	−0.13	0.39	−0.13	0.22
Oily fish	−0.19	0.38	−0.01	−0.06
Vegetables not raw	−0.23	0.34	0.22	0.00
Fruit juice including smoothies	0.07	0.27	0.11	0.00
Nuts and seeds	0.02	0.27	−0.06	0.13
Skimmed milk	−0.03	0.25	−0.06	0.02
White fish coated or fried	−0.07	−0.25	0.04	0.06
Other margarine, fats, and oils	−0.04	0.21	0.09	0.03
Other bread	0.02	0.21	0.05	−0.05
Other white fish, shellfish, and fish dishes	−0.06	0.20	−0.12	−0.09
Other milk and cream	0.03	0.19	0.04	−0.04
Brown, granary, and wheat germ bread	0.01	0.15	−0.03	0.00
PUFA margarine and oils	−0.05	0.09	0.04	0.02
Other potatoes, potato salads and dishes	−0.23	0.14	0.47	0.03
Sauces, pickles, and gravies	0.06	0.18	0.47	−0.05
White bread	0.30	−0.27	0.44	0.13
Butter	−0.09	−0.02	0.35	0.04
Sugar, preserves, and sweet spreads	−0.10	−0.26	0.34	0.29
Bacon and ham	0.18	0.05	0.34	0.09
Beer, lager, cider, and perry	0.22	−0.07	0.29	−0.16
Meat pies and pastries	0.09	−0.14	0.28	0.04
Sausages	0.06	−0.11	0.27	0.03
Whole milk	−0.07	−0.18	0.25	−0.03
Pork and dishes	−0.01	0.03	0.21	−0.05
Eggs and egg dishes	−0.07	0.16	0.20	−0.15
Other meat and meat products	0.02	0.05	0.18	−0.02
Beef, veal, and dishes	0.06	−0.10	−0.15	−0.004
Liver and dishes	−0.08	−0.002	0.10	−0.04
Low fat spread not polyunsaturated	−0.01	0.03	−0.03	−0.03
Biscuits	0.04	0.04	−0.13	0.47
Semi skimmed milk	−0.17	−0.12	−0.03	0.43
Chocolate confectionery	0.35	0.13	0.02	0.40
Buns, cakes, pastries, and fruit pies	−0.12	0.11	0.19	0.38
Wine	0.03	0.28	0.13	−0.31
Puddings	−0.13	0.01	0.12	0.28
Cheese	0.01	0.15	0.12	0.28
Ice cream	0.03	0.07	−0.002	0.24
Reduced fat spread not polyunsaturated	0.12	−0.14	0.19	0.21
Spirits and liqueurs	0.17	0.08	0.15	−0.20
Reduced fat spread polyunsaturated	−0.08	−0.13	0.01	0.16
Other breakfast cereals	0.001	−0.04	0.001	0.15
Dry weight beverages	−0.07	−0.04	−0.06	0.09
Lamb and dishes	−0.02	−0.02	0.03	−0.08

SFFFD: ‘Snacks, fast food, fizzy drinks’; FVOF: ‘Fruit, vegetables, oily fish’; MPB: ‘Meat, potatoes, beer’; SFD: ‘Sugary foods, dairy’.

**Table 4:** Food variables characterising each dietary pattern (NDNS 2008–2012).

Dietary Pattern Label	Foods with Moderate/Strong Positive Factor Loadings (≥0.25)	Foods with Moderate/Strong Negative Factor Loadings (≤−0.25)
‘Snacks, fast food, fizzy drinks’ (SFFFD)	Soft drinks (not low calorie) Crisps and savoury snacks Soft drinks (low calorie) Coated chicken and turkey Burgers and kebabs Chocolate confectionery Chips (fried), roast potatoes, and potato products White bread Sugar confectionery Pasta, rice, and other cereals	Tea, coffee, and water High fibre breakfast cereals Fruit Wholemeal bread
‘Fruit, vegetables, oily fish’ (FVOF)	Fruit Salad and other raw vegetables Yoghurt, fromage frais, and dairy deserts Oily fish Vegetables not raw Fruit juice including smoothies Wine Nuts and seeds Skimmed milk	Chips (fried), roast potatoes, and potato products White bread Sugar, preserves, and sweet spreads White fish, coated or fried
‘Meat, potatoes, beer’ (MPB)	Other potatoes, potato salads and dishes Savoury sauces, pickles, gravies, and condiments White bread Butter Sugar, preserves, and sweet spreads Bacon and ham Beer, lager, cider, and perry Meat pies and pastries Sausages Whole milk	
‘Sugary food, dairy’ (SFD)	Biscuits Semi skimmed milk Chocolate confectionery Buns, cakes, pastries, and fruit pies Sugars, preserves, and sweet spreads Puddings Cheese Tea, coffee, and water	Wine

**Table 5:** Main effects (95% confidence interval (CI)) of each dietary pattern for sociodemographic characteristics and lifestyle factors.

Sample Characteristics (*n*)	Snacks, Fast Food, Fizzy Drinks (SFFFD)		Fruit, Veg, Oily Fish (FVOF)		Meat, Potatoes, Beer (MPB)		Sugary Foods, Dairy (SFD)	
	Coefficient (95% CI)	*p*	Coefficient (95% CI)	*p*	Coefficient (95% CI)	*p*	Coefficient (95% CI)	*p*
*Sex*								
Male (711)	0.24 (0.16, 0.33)	<0.001	−0.08 (−0.08, −0.08)	<0.001	0.63 (0.54, 0.72)	<0.001	0.19 (0.10, 0.29)	<0.001
Female (914)	*Reference* *							
*Age year (2083)*	−0.03 (−0.03, −0.03)	<0.001	0.002 (0.002, 0.002)	<0.001	0.01 (0.004, 0.01)	<0.001	0.003 (<0.001, 0.01)	<0.001
*Ethnicity*								
Non-white (1495)	−0.14 (0.06, −0.27)	0.72	0.22 (0.05, −0.39)	0.01	−0.56 (−0.73, −0.39)	<0.001	−0.46 (−0.65, −0.30)	<0.001
White (130)	*Reference* *							
*National Statistics Socio-economic Classification Group (NSSEC)*								
Higher managerial and professional (260)	0.12 (−0.20, 0.30)	0.46	0.25 (0.24, 0.27)	<0.001	0.03 (−0.33, 0.37)	0.89	0.45 (0.09, 0.81)	0.02
Lower managerial and professional (453)	0.21 (−0.10, 0.51)	0.18	0.14 (0.13, 0.15)	<0.001	−0.04 (−0.37, 0.30)	0.83	0.43 (0.08, 0.78)	0.02
Intermediate occupations ^1^ (149)	0.21 (−0.11, 0.53)	0.20	−0.02 (−0.03, −0.01)	0.002	0.04 (−0.31, 0.40)	0.81	0.38 (0.01, 0.75)	0.04
Small employers (165)	0.05 (−0.27, 0.34)	0.76	−0.03 (−0.04, −0.02)	<0.001	0.25 (−0.10, 0.61)	0.16	0.37 (0.007, 0.74)	0.05
Low supervisory and technical occupations (160)	0.16 (−0.16, 0.47)	0.33	−0.12 (−0.14, −0.11)	<0.001	0.09 (−0.27, 0.44)	0.63	0.53 (0.16, 0.89)	0.005
Semi-routine occupations ^2^ (228)	0.27 (−0.4, 0.56)	0.09	−0.11 (−0.12, −0.10)	<0.001	0.21 (−0.13, 0.56)	0.23	0.52 (0.16, 0.87)	0.004
Routine occupations ^3^ (177)	0.20 (−0.11, 0.52)	0.21	−0.36 (−0.37, −0.35)	<0.001	0.20 (−0.16, 0.55)	0.27	0.25 (−0.11, 0.62)	0.17
Never worked (33)	*Reference* *							
*Smoking status*								
Smoker (377)	0.16 (0.06, 0.26)	0.002	−0.37 (−0.38, −0.37)	<0.001	0.21 (0.10, 0.33)	<0.001	−0.21 (−0.33, −0.10)	<0.001
Non-smoker (1248)	*Reference* *							
*Household income (1777)*	7.627 × 10^−7^ (−1.207 × 10, 2.733 × 10^−6^)	0.45	7.112 × 10^−6^ (7.044 × 10^−6^, 7.181 × 10^−6^)	<0.001	−9.357 × 10^−7^ (−3.146 × 10^−6^, 1.274 × 10^−6^)	0.41	−2.192 × 10^−6^ (−4.471 × 10^−6^, 8.576 × 10^−8^)	0.06
*Body Mass Index (BMI) (1902)*	0.13 (0.005, 0.02)	0.01	−0.002 (−0.002, −0.001)	<0.001	−0.003 (−0.12, 0.005)	0.42	−0.02 (−0.03, −0.01)	<0.001

* Data that are not reported were set to zero for the reference category. ^1^ Intermediate occupations are positions in, for example, clerical, sales, service, and intermediate technical occupations that do not involve general planning or supervisory powers. Positions in this group are intermediate in terms of employment regulation, i.e., they combine elements of both the service relationship and the labour contract; ^2^ Semi-routine occupations are positions in, for example, sales, service, clerical, or operative occupations with a labour contract that is typified by being short term or the direct exchange of money for effort. The work involved requires at least some element of employee discretion; ^3^ Routine occupations are positions with a basic labour contract, in which employees are engaged in routine work [63].

**Table 6:** Main effects (95% confidence intervals) of dietary patterns for self-reported nutrient intakes and biomarkers.

	Snacks, Fast Foods, Fizzy Drinks	Fruit, Vegetables, Oily Fish	Meat, Potatoes, Beer	Sugary Foods, Dairy
	Coefficients (95% Confidence Intervals), * *p* < 0.05, ** *p* < 0.01, *** *p* < 0.001			
*Nutrient intake*				
Vitamin C per 1000 kcal	−0.01 (−0.006, −0.004) ***	0.01 (0.011, 0.014) ***	−0.003 (−0.004, −0.002) ***	−0.003 (−0.004, −0.002) ***
Vitamin D per 1000 kcal	−0.20 (−0.23, −0.16) ***	0.17 (0.133, 0.20) ***	0.01 (−0.03, 0.41)	−0.10 (−0.13, −0.6) ***
Vitamin E per 1000 kcal	−0.04 (−0.05, −0.02) ***	0.08 (0.07, 0.10) ***	−0.06 (−0.7, −0.4) ***	−0.02 (−0.4, −0.01) **
Vitamin B_6_ per 1000 kcal	−0.19 (−0.27, −0.11) **	0.09 (0.01, 0.18) *	−0.03 (−0.16, 0.05)	−0.40 (−0.48, −0.32) ***
Vitamin B_12_ per 1000 kcal	−0.10 (−0.12, −0.08) ***	0.04 (0.02, 0.05) ***	−0.01 (−0.03, 0.01)	−0.05 (−0.07, −0.03) ***
Iron per 1000 kcal	−0.12 (−0.14, −0.10) ***	0.11 (0.09, 0.14) ***	−0.08 (−0.10, −0.06) ***	−0.14 (−0.16, −0.12) ***
Folate per 1000 kcal	−0.01 (−0.006, −0.005) ***	0.003 (0.003, 0.004) ***	−0.001 (−0.001, 0.001)	−0.003 (−0.004, −0.002) ***
Magnesium per 1000 kcal	−0.01 (−0.01, −0.008) ***	0.01 (0.009, 0.011) ***	−0.01 (−0.006, −0.004) ***	−0.01 (−0.006, −0.004) ***
% Food Energy NMES	0.05 (0.04, 0.05) ***	−0.01 (0.21, 0.007) ***	0.02 (0.02, 0.03) ***	0.03 (0.02, 0.03) ***
% Food Energy Sat Fat	−0.12 (−0.024, 0.001)	−0.04 (−0.05, −0.02) ***	0.08 (0.07, 0.09) ***	0.06 (0.05, 0.07) ***
% Food Energy Total Fat	0.01 (0.01, 0.02) ***	−0.01 (−0.02, −0.002) **	0.04 (0.03, 0.05) ***	0.01 (0.01, −0.02) ***
% Food Energy *n*-3 PUFA	−0.21 (−0.30, −0.12) ***	0.45 (0.36, 0.54) ***	0.08 (−0.01, 0.17)	−0.31 (−0.40, −0.22) ***
% Food Energy *n*-6 PUFA	0.06 (0.03, 0.08) ***	0.03 (0.002, 0.06) *	−0.004 (−0.03, 0.02)	−0.04 (−0.07, −0.01) **
% Food Energy Starch	0.01 (0.001, 0.02) *	−0.04 (−0.05, −0.03) ***	−0.02 (−0.03, -0.01) ***	−0.02 (−0.03, −0.01) ***
Non-starch polysaccharides (fibre)	−0.03 (−0.04, −0.02) ***	0.11 (0.10, 0.12) ***	0.03 (0.02, 0.04) ***	0.07 (0.06, 0.08) ***
*Nutrient level from biomarkers*				
Vitamin C	−0.004 (−0.007, −0.001) *	0.02 (0.01, 0.02) ***	−0.004 (−0.01, −0.001) *	0.001 (−0.002, 0.004)
25-Hydroxy Vitamin D	−0.002 (−0.004, 0.001)	0.01 (0.004, 0.009) ***	−0.001 (−0.003, 0.002)	0.0004 (−0.002, 0.003)
Retinol (Vitamin A)	−0.10 (−0.20, 0.004)	0.13 (0.02, 0.23) *	0.12 (−0.001, 0.21)	−0.18 (−0.29, −0.08) ***
Ferritin (iron)	4.908 × 10^−5^ (−0.001, −0.001)	0.001 (5.153 × 10^−5^, 0.001) *	0.001 (0.001, 0.002) ***	−0.001 (−0.001, −3.55) *
Triglycerides	0.01 (−0.05, 0.08)	−0.06 (−0.13, 0.002)	0.13 (0.06, 0.19) ***	−0.02 (−0.09, 0.04)
Total cholesterol	−0.07 (−0.13, −0.02) **	0.04 (−0.01, 0.10)	−0.03 (−0.08, 0.03)	−0.07 (−0.13, −0.02)
Urinary sodium	0.01 (0.005, 0.008) ***	−0.01 (−0.006, −0.003) ***	0.002 (0.0004, 0.003) *	−0.002 (−0.003, 0.0001) *
Total carotenoids	−0.08 (−0.15, −0.02) *	0.32 (0.26, 0.38) ***	−0.16 (−0.23, −0.09) ***	−0.03 (−0.09, 0.04)

**Table 7:** Main effects (95% confidence intervals, *p* < 0.001) of the NDQS and dietary patterns (unadjusted and adjusted).

Dietary Patterns	Coefficient (Unadjusted)	CI (95%) Lower	Upper	Coefficient (Adjusted)	CI (95%) Lower	Upper
Snacks, Fast Food, Fizzy Drinks (SFFFD)	−3.727	−4.205	−3.249	−3.31	−3.97	−2.64
Fruit, Vegetables, Oily Fish (FVOF)	4.514	4.037	4.992	3.79	3.19	4.40
Meat, Potatoes, Beer (MPB)	−1.183	−1.660	−0.705	−1.76	−2.43	−1.09
Sugary Foods, Dairy (SFD)	2.425	1.948	2.903	1.31	0.69	1.93

## References

[1] Willett W.C. (2013). Nutritional Epidemiology.

[2] Hu F.B. (2002). Dietary pattern analysis: A new direction in nutritional epidemiology. Curr. Opin. Lipidol..

[3] Craig W.J. (1997). Phytochemicals: Guardians of our health. J. Am. Diet. Assoc..

[4] Liu R.H. (2004). Potential synergy of phytochemicals in cancer prevention: Mechanism of action. J. Nutr..

[5] Mertz W. (1984). Foods and nutrients. J. Am. Diet. Assoc..

[6] Kant A.K. (1996). Indexes of overall diet quality: A review. J. Am. Diet. Assoc..

[7] Cunha D.B., Varnier R.M., de Almeda R., Pereira R.A. (2010). A comparison of three statistical methods applied in the identification of eating patterns. Cadernos de Saúde Pública.

[8] Willett W.C., McCullough M.L. (2008). Dietary pattern analysis for the evaluation of dietary guidelines. Asia Pac. J. Clin. Nutr..

[9] Trichopoulou A., Costacou T., Bamia C., Trichopoulos D. (2003). Adherence to a mediterranean diet and survival in a Greek population. N. Engl. J. Med..

[10] Esposito K., Maiorino M.I., Ceriello A., Giugliano D. (2010). Prevention and control of type 2 diabetes by mediterranean diet: A systematic review. Diabetes Res. Clin. Pract..

[11] De Lorgeril M., Salen P. (2006). The mediterranean-style diet for the prevention of cardiovascular diseases. Public Health Nutr..

[12] Kennedy E.T., Ohls J., Carlson S., Fleming K. (1995). The healthy eating index: Design and applications. J. Am. Diet. Assoc..

[13] Guo X., Warden B.A., Paeratakul S., Bray G.A. (2004). Healthy eating index and obesity. Eur. J. Clin. Nutr..

[14] Weinstein S.J., Vogt T.M., Gerrior S.A. (2004). Healthy eating index scores are associated with blood nutrient concentrations in the third national health and nutrition examination survey. J. Am. Diet. Assoc..

[15] Shah B.S., Freeland-Graves J.H., Cahill J.M., Lu H., Graves G.R. (2010). Diet quality as measured by the healthy eating index and the association with lipid profile in low-income women in early postpartum. J. Am. Diet. Assoc..

[16] Lin P.H., Aickin M., Champagne C., Craddick S., Sacks F.M., McCarron P., Most-Windhauser M.M., Rukenbrod F., Haworth L., Dash-Sodium Collaborative Research Group (2003). Food group sources of nutrients in the dietary patterns of the Dash-Sodium trial. J. Am. Diet. Assoc..

[17] Sacks F.M., Svetkey L.P., Vollmer W.M., Appel L.J., Bray G.A., Harsha D., Obarzanek E., Conlin P.R., Miller E.R., Simons-Morton D.G. (2001). Effects on blood pressure of reduced dietary sodium and the dietary approaches to stop hypertension (DASH) diet. Dash-sodium collaborative research group. N. Engl. J. Med..

[18] Reedy J., Wirfalt E., Flood A., Mitrou P.N., Krebs-Smith S.M., Kipnis V., Midthune D., Leitzmann M., Hollenbeck A., Schatzkin A. (2010). Comparing 3 dietary pattern methods—Cluster analysis, factor analysis, and index analysis—With colorectal cancer risk: The NIH-AARP diet and health study. Am. J. Epidemiol..

[19] Schulze M.B., Hu F.B. (2002). Dietary patterns and risk of hypertension, type 2 diabetes mellitus, and coronary heart disease. Curr. Atheroscler. Rep..

[20] Osler M., Helms Andreasen A., Heitmann B., Hoidrup S., Gerdes U., Morch Jorgensen L., Schroll M. (2002). Food intake patterns and risk of coronary heart disease: A prospective cohort study examining the use of traditional scoring techniques. Eur. J. Clin. Nutr..

[21] Greenwood D.C., Cade J.E., Draper A., Barrett J.H., Calvert C., Greenhalgh A. (2000). Seven unique food consumption patterns identified among women in the UK women’s cohort study. Eur. J. Clin. Nutr..

[22] Lin H., Bermudez O.I., Tucker K.L. (2003). Dietary patterns of Hispanic elders are associated with acculturation and obesity. J. Nutr..

[23] Johnson L., Mander A.P., Jones L.R., Emmett P.M., Jebb S.A. (2008). Energy-dense, low-fiber, high-fat dietary pattern is associated with increased fatness in childhood. Am. J. Clin. Nutr..

[24] Ambrosini G.L., Emmett P.M., Northstone K., Howe L.D., Tilling K., Jebb S.A. (2012). Identification of a dietary pattern prospectively associated with increased adiposity during childhood and adolescence. Int. J. Obes..

[25] Northstone K., Emmett P.M. (2010). Dietary patterns of men in ALSPAC: Associations with socio-demographic and lifestyle characteristics, nutrient intake and comparison with women’s dietary patterns. Eur. J. Clin. Nutr..

[26] Northstone K., Joinson C., Emmett P., Ness A., Paus T. (2012). Are dietary patterns in childhood associated with IQ at 8 years of age? A population-based cohort study. J. Epidemiol. Community Health.

[27] Feinstein L., Sabates R., Sorhaindo A., Rogers I., Herrick D., Northstone K., Emmett P. (2008). Dietary patterns related to attainment in school: The importance of early eating patterns. J. Epidemiol. Community Health.

[28] Mishra G.D., dos Santos Silva I., McNaughton S.A., Stephen A., Kuh D. (2011). Energy intake and dietary patterns in childhood and throughout adulthood and mammographic density: Results from a British prospective cohort. Cancer Causes Control.

[29] Shaheen S.O., Jameson K.A., Syddall H.E., Aihie Sayer A., Dennison E.M., Cooper C., Robinson S.M., Hertfordshire Cohort Study Group (2010). The relationship of dietary patterns with adult lung function and COPD. Eur. Respir. J..

[30] Poslusna K., Ruprich J., de Vries J.H., Jakubikova M., van’t Veer P. (2009). Misreporting of energy and micronutrient intake estimated by food records and 24 hour recalls, control and adjustment methods in practice. Br. J. Nutr..

[31] Archer E., Hand G.A., Blair S.N. (2013). Validity of U.S. Nutritional surveillance: National health and nutrition examination survey caloric energy intake data, 1971–2010. PLoS ONE.

[32] Freedman L.S., Commins J.M., Moler J.E., Arab L., Baer D.J., Kipnis V., Midthune D., Moshfegh A.J., Neuhouser M.L., Prentice R.L. (2014). Pooled results from 5 validation studies of dietary self-report instruments using recovery biomarkers for energy and protein intake. Am. J. Epidemiol..

[33] Schoeller D., Archer E., Dawson J.A., Heymsfield S. (2015). Implausible results from the use of invalid methods. J. Nutr..

[34] Schoeller D.A., Thomas D., Archer E., Heymsfield S.B., Blair S.N., Goran M.I., Hill J.O., Atkinson R.L., Corkey B.E., Foreyt J. (2013). Self-report-based estimates of energy intake offer an inadequate basis for scientific conclusions. Am. J. Clin. Nutr..

[35] Subar A.F., Freedman L.S., Tooze J.A., Kirkpatrick S.I., Boushey C., Neuhouser M.L., Thompson F.E., Potischman N., Guenther P.M., Tarasuk V. (2015). Addressing current criticism regarding the value of self-report dietary data. J. Nutr..

[36] Freedman L.S., Schatzkin A., Midthune D., Kipnis V. (2011). Dealing with dietary measurement error in nutritional cohort studies. J. Natl. Cancer Inst..

[37] Hu F.B., Stampfer M.J., Rimm E., Ascherio A., Rosner B.A., Spiegelman D., Willett W.C. (1999). Dietary fat and coronary heart disease: A comparison of approaches for adjusting for total energy intake and modeling repeated dietary measurements. Am. J. Epidemiol..

[38] Cade J.E. (2017). Measuring diet in the 21st century: Use of new technologies. Proc. Nutr. Soc..

[39] Cade J.E., Warthon-Medina M., Albar S., Alwan N.A., Ness A., Roe M., Wark P.A., Greathead K., Burley V.J., Finglas P. (2017). Diet@net: Best practice guidelines for dietary assessment in health research. BMC Med..

[40] Black A.E. (1996). Physical activity levels from a meta-analysis of doubly labeled water studies for validating energy intake as measured by dietary assessment. Nutr. Rev..

[41] Hill R.J., Davies P.S. (2001). The validity of self-reported energy intake as determined using the doubly labelled water technique. Br. J. Nutr..

[42] Prentice R.L., Huang Y., Tinker L.F., Beresford S.A., Lampe J.W., Neuhouser M.L. (2009). Statistical aspects of the use of biomarkers in nutritional epidemiology research. Stat. Biosci..

[43] Willet W., Rimm E.B., Hu F.B. (2017). Response to E Archer. Am. J. Clin. Nutr..

[44] Hebert J.R., Hurley T.G., Steck S.E., Miller D.R., Tabung F.K., Peterson K.E., Kushi L.H., Frongillo E.A. (2014). Considering the value of dietary assessment data in informing nutrition-related health policy. Adv. Nutr..

[45] Public Health England (2014). Programme User Guide for UK Core Sample Data.

[46] NatCen Social Research MHNRaUCL (2015). National Diet and Nutrition Survey 1–4, 2008/09–2011/12.

[47] Whitton C., Nicholson S.K., Roberts C., Prynne C.J., Pot G.K., Olson A., Fitt E., Cole D., Teucher B., Bates B. (2011). National diet and nutrition survey: UK food consumption and nutrient intakes from the first year of the rolling programme and comparisons with previous surveys. Br. J. Nutr..

[48] Newby P.K., Tucker K.L. (2004). Empirically derived eating patterns using factor or cluster analysis: A review. Nutr. Rev..

[49] Field A. (2009). Discovering Statistics Using Spss.

[50] Northstone K., Emmett P., Rogers I. (2008). Dietary patterns in pregnancy and associations with socio-demographic and lifestyle factors. Eur. J. Clin. Nutr..

[51] North K., Emmett P. (2000). Multivariate analysis of diet among three-year-old children and associations with socio-demographic characteristics. The Avon Longitudinal Study of Pregnancy and Childhood (ALSPAC) Study Team. Eur. J. Clin. Nutr..

[52] Pot G.K., Stephen A.M., Dahm C.C., Key T.J., Cairns B.J., Burley V.J., Cade J.E., Greenwood D.C., Keogh R.H., Bhaniani A. (2014). Dietary patterns derived with multiple methods from food diaries and breast cancer risk in the UK dietary cohort consortium. Eur. J. Clin. Nutr..

[53] Bennette C., Vickers A. (2012). Against quantiles: Categorization of continuous variables in epidemiologic research, and its discontents. BMC Med. Res. Methodol..

[54] Katz M.H. (2011). Multivariable Analysis: A Practical Guide for Clinicians and Public Health Researchers.

[55] Committee on Medical Aspects of Food Policy (1991). Dietary reference values for food energy and nutrients for the United Kingdom. Report of the panel on dietary reference values of the committee on medical aspects of food policy. Rep. Health Soc. Subj..

[56] Scientific Advisory Committee on Nutrition (2011). Dietary Reference Values for Food Energy and Nutrients for the United Kingdom.

[57] England P.H. (2016). Government Dietary Recommendations.

[58] Public Health England (2014). National Diet and Nutrition Survey Results from Years 1, 2, 3 and 4 (Combined) of the Rolling Programme (2008/09–2011/12).

[59] Public Health England 3.8 Million People in England Now Have Diabetes. https://www.gov.uk/government/news/38-million-people-in-england-now-have-diabetes.

[60] Public Health England (2015). Sugar Reduction: The Evidence for Action.

[61] Rayner M., Scarborough P. (2005). The burden of food related ill health in the UK. J. Epidemiol. Community Health.

[62] Roberts K.E. (2017). An Investigation of Dietary Patterns in UK Adults as a Method for developing a Brief Diet Quality Assessment Tool. Ph.D. Thesis.

[63] Office of National Statistics (2005). The National Statistics Socio-Economic Classification User Manual.

[64] Markussen M.S., Veierod M.B., Sakhi A.K., Ellingjord-Dale M., Blomhoff R., Ursin G., Andersen L.F. (2015). Evaluation of dietary patterns among Norwegian postmenopausal women using plasma carotenoids as biomarkers. Br. J. Nutr..

[65] Marmot M., Bell R. (2012). Fair society, healthy lives. Public Health.

[66] Hamer M., Mishra G.D. (2010). Dietary patterns and cardiovascular risk markers in the UK low income diet and nutrition survey. Nutr. Metab. Cardiovasc. Dis..

[67] Northstone K., Emmett P.M., Rogers I. (2008). Dietary patterns in pregnancy and associations with nutrient intakes. Br. J. Nutr..

[68] McCourt H.J., Draffin C.R., Woodside J.V., Cardwell C.R., Young I.S., Hunter S.J., Murray L.J., Boreham C.A., Gallagher A.M., Neville C.E. (2014). Dietary patterns and cardiovascular risk factors in adolescents and young adults: The northern Ireland young hearts project. Br. J. Nutr..

[69] McCann S.E., Marshall J.R., Brasure J.R., Graham S., Freudenheim J.L. (2001). Analysis of patterns of food intake in nutritional epidemiology: Food classification in principal components analysis and the subsequent impact on estimates for endometrial cancer. Public Health Nutr..

[70] Gibson S., Ashwell M. (2011). Dietary patterns among British adults: Compatibility with dietary guidelines for salt/sodium, fat, saturated fat and sugars. Public Health Nutr..

